# A New Sign of Basilar Meningitis

**Published:** 1904-05-07

**Authors:** 


### A NEW SIGN OF BASILAR MENINGITIS.

Under this heading Dr. Squires[1] describes a sign which he states has been invariably present in cases of tuberculous meningitis, frequently so early as the fourth or fifth day of the illness. The sign consists of a rhythmical contraction and dilatation of the pupil which ensues when certain manipulations are performed on the child's head and neck. The method of obtaining the sign is as follows:— "Place the child's head face upward between the knees of the physician, with the body of the child supported on the bed, table, or lap of the nurse. Grasp the sides of the child's head with each hand and produce gradual and forcible extension of the head on the spinal column. As the head is brought back in this extension the pupils will be seen to commence to dilate simultaneously with the commencement of extension, the more extreme the extension the more the dilatation. Now upon flexion the pupils contract so that when the chin is forcibly brought to the manubrium the pupils are well closed up. This can be done several times a minute, and each time the pupillary phenomenon will be repeated. In view of the importance of an early recognition and treatment of this disease it will be interesting to observe whether the sign ultimately proves to be of so much diagnostic value as Dr. Squires anticipates.

[1] Medical Record, March 26.

